# High Resolution Genomic Scans Reveal Genetic Architecture Controlling Alcohol Preference in Bidirectionally Selected Rat Model

**DOI:** 10.1371/journal.pgen.1006178

**Published:** 2016-08-04

**Authors:** Chiao-Ling Lo, Amy C. Lossie, Tiebing Liang, Yunlong Liu, Xiaoling Xuei, Lawrence Lumeng, Feng C. Zhou, William M. Muir

**Affiliations:** 1 Indiana Alcohol Research Center, Indiana University School of Medicine, Indianapolis, Indiana, United States of America; 2 Department of Anatomy & Cell Biology, Indiana University School of Medicine, Indianapolis, Indiana, United States of America; 3 Department of Animal Sciences, Purdue University, West Lafayette, Indiana, United States of America; 4 Department of Medicine, Indiana University School of Medicine, Indianapolis, Indiana, United States of America; 5 Department of Medical Genetics, Indiana University School of Medicine, Indianapolis, Indiana, United States of America; 6 Department of Biochemistry, Indiana University School of Medicine, Indianapolis, Indiana, United States of America; 7 Stark Neuroscience Research Institute, Indiana University School of Medicine, Indianapolis, Indiana, United States of America; Georgia Institute of Technology, UNITED STATES

## Abstract

Investigations on the influence of nature vs. nurture on Alcoholism (Alcohol Use Disorder) in human have yet to provide a clear view on potential genomic etiologies. To address this issue, we sequenced a replicated animal model system bidirectionally-selected for alcohol preference (AP). This model is uniquely suited to map genetic effects with high reproducibility, and resolution. The origin of the rat lines (an 8-way cross) resulted in small haplotype blocks (HB) with a corresponding high level of resolution. We sequenced DNAs from 40 samples (10 per line of each replicate) to determine allele frequencies and HB. We achieved ~46X coverage per line and replicate. Excessive differentiation in the genomic architecture between lines, across replicates, termed signatures of selection (SS), were classified according to gene and region. We identified SS in 930 genes associated with AP. The majority (50%) of the SS were confined to single gene regions, the greatest numbers of which were in promoters (284) and intronic regions (169) with the least in exon's (4), suggesting that differences in AP were primarily due to alterations in regulatory regions. We confirmed previously identified genes and found many new genes associated with AP. Of those newly identified genes, several demonstrated neuronal function involved in synaptic memory and reward behavior, e.g. ion channels (*Kcnf1*, *Kcnn3*, *Scn5a*), excitatory receptors (*Grin2a*, *Gria3*, *Grip1)*, neurotransmitters (*Pomc*), and synapses (*Snap29*). This study not only reveals the polygenic architecture of AP, but also emphasizes the importance of regulatory elements, consistent with other complex traits.

## Author Summary

Alcohol Used Disorder (AUD) or Alcoholism extracts a great societal cost in terms of human suffering. Understanding the genetic basis is critical to comprehend, treat and prevent this disease, but difficult in humans, as choice is influenced by nature and nurture. To discover its genetic basis, we used an animal model system that controlled for genetic and non-genetic factors through randomization, study replication, long-term divergent selection, and a controlled environment. We conducted whole genome sequencing in breeds that were either compulsive excessive drinkers or completely abstinent. We discovered consistent alterations in several genes and neurological pathways previously unassociated with alcoholism. These results strengthened our understanding of the genetic basis of alcoholism and revealed potential genetic- and neurological-based treatments.

## Introduction

The quest to discover the underlying genetic etiology that contributes to alcoholism (a.k.a. Alcohol Use Disorder, AUD) is an ultimate goal for understanding, preventing and treating alcoholism caused by inherited risk factors. Genomic investigations in humans have yet to determine the genetic causes of this disease due to a number of challenges, including partial or complete confounding of family history with familial drinking behaviors, drinking variability and non-genetic (e.g. social, economical and cultural) factors. Genomic analyses in humans, including genome-wide association studies (GWAS), account for a small proportion of the total genetic variance associated with AUD [1–4]. In addition, reproducibility of results has become a major concern [5]. To address these issues, an animal model with heterogeneous origins [6], combined with replication and randomization (see below), was utilized with the expectation that the model will gain insights into the human condition.

The high (HAD) and low (LAD) alcohol drinking rat lines constitute one of the most extensively characterized rodent models of human AUD [7]. These bi-directionally selected (>40 generations) and replicated lines [8] exhibit a wide range of alcohol preferences (AP) and other alcohol-related endophenotypes that recapitulate human AUD [7, 9]. The HAD/LAD lines were derived from the NIH heterogeneous stock (NIH-HS) [10] and encompass genetic variation from eight inbred lines. The HAD/LAD demonstrate a major heritable component for AP, providing an attractive model to expose the genetic and neurobiological bases underlying AUDs.

The AP trait is characterized by heightened alcohol seeking behavior driven by the reinforcing action and other effects of ethanol on the brain. HAD rats show the reinforcing effects through performing an operant response for access to alcohol and attain pharmacologically relevant blood alcohol concentrations (BACs; 50 to 150 mg/dL) in under 24 hours [7]. HAD animals display an alcohol deprivation effect after repeated cycles of access to a single concentration of alcohol and an increased response when multiple concentrations of ethanol are used. In contrast, the LAD lines exhibit none of these phenotypes [7]. Although previous studies from our group identified several candidate genes associated with AP on Chrs 5, 10, 12 and 16 in HAD rats [11–13], the QTL resolving power precluded fine mapping of the causative genes.

In this study we employed whole genome sequencing to identify regions of excessive differentiation associated with AP using the population stratification index (*Fst*). The *Fst* identifies genes and gene regions under selection by finding genomic regions with an excessive degree of differentiation [14–17]. While this approach has been tried in a number of studies [18–20], most were inconclusive due to issues related to demography, lack of replication [21] or large LD blocks. We eliminated most of these issues by using a heterogeneous, bi-directionally-selected and replicated animal model that enabled us to detect genomic signatures of selection (SS) for AP in the HAD/LAD lines including HAD1/LAD1 lines and replicate HAD2/LAD2 lines. We identified specific genes and in many cases regions within those genes under selection, allowing insight into the importance of regulatory *vs*. coding regions in the evolution of phenotypes associated with AP. Our findings show SS in genes previously predicted in the human population and identify new genes that are functionally relevant to the plasticity of the reward mechanism indicative of AP.

## Results

### SNPs, Linkage Disequilibrium (LD), and Haplotype Blocks (HB)

**Fig 1:**
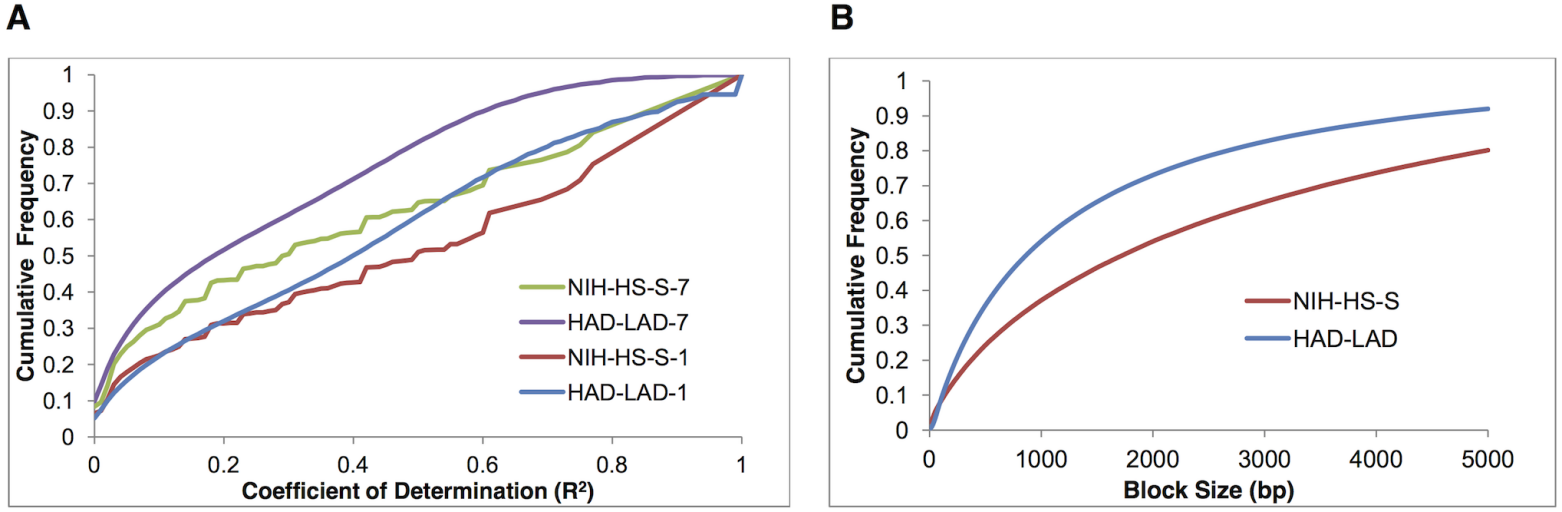
A. Cumulative frequency distribution of R^2^ by line and distance. For pairs of polymorphic loci, either 7 SNPs apart (NIH-HS-7, HAD-LAD-7) or adjacent (NIH-HS-1, HAD-LAD-1). Genotype frequencies in the NIH-HS was quantified by combining genotypes from the sequence data of each of the 8 parent lines into an *in silico* NIH-HS-S and estimating haplotype frequencies. For the NIH-HS-S population, the R^2^ values were based on ~2.9 million pairs of jointly polymorphic loci, each with a minimum minor allele frequency of 10%. For the HAD-LAD joint population, the R^2^ values were based on ~3.5 million pairs of jointly polymorphic loci, each with a minimum minor allele frequency of 10%. B. Distribution of haplotype blocks (HB) sizes across the genome for these lines defined using the 4-gamete rule [22].

Following whole genome sequencing, variants (SNPs) were called using GATK as described in Methods-*Sequence assembly and variant calls*. There were 6,585,606 SNPs identified when combined across replicates. Allele frequencies and *Fst* statistics were determined from these SNPs. While sequencing errors were filtered, any remaining are expected to have only minor effects on the metric we used for finding SS (see Discussion-*Sequencing Errors*, *Window Size*, *LD and Haplotype Blocks*). From these SNPs, we found that the LD_25 (the genomic distance for which the R^2^ < .25, see Methods-*Detecting Signatures of selection (SS)*), combined across replicates, was approximately 1.4kb and included 7 SNPs on average. Consequently, the sliding window used to calculate the running average of the *Fst* statistic was based on 7 adjacent SNPs, 3 on either side of the central or focal position. Remarkably, from the cumulative frequency distribution of R^2^ and for pairs of loci 7 SNPs apart (Fig 1A), approximately 55.6% had an R^2^ less than .25 and 81.4% had an R^2^ less than 0.50, indicating that the majority of variation between loci at this distance is independent of linkage.

Similarly, the average haplotype block (HB) length was small (Fig 1B). We defined HB using the Four Gamete Rule (FGR) [23] (see Methods-*Genotypes*, *Allele frequencies*, *Linkage Disequilibrium*, *and Haplotype Blocks*). For comparison with the base NIH-HS-S population, we used a restriction that the least frequent gamete (Minor Gamete Frequency, MGF) be > 6.25% and resulted in a median HB size of 870bp in the HAD/LAD lines. Using the same MGF, the median HB size for the NIH-HS-S base population, was 1730bp (Fig 1B), showing that initial HB length was small and 60+ generations of random mating reduced the interval by ~50%. However, for identification of SS associated with gene regions, we used a more conservative MGF = 9%. For this value of MGF, we observed a near continuous spine of HB (Haploview), and the median HB length was 1.48kb, slightly larger than the LD_25 based on R^2^.

### Signatures of Selection (SS)

**Fig 2:**
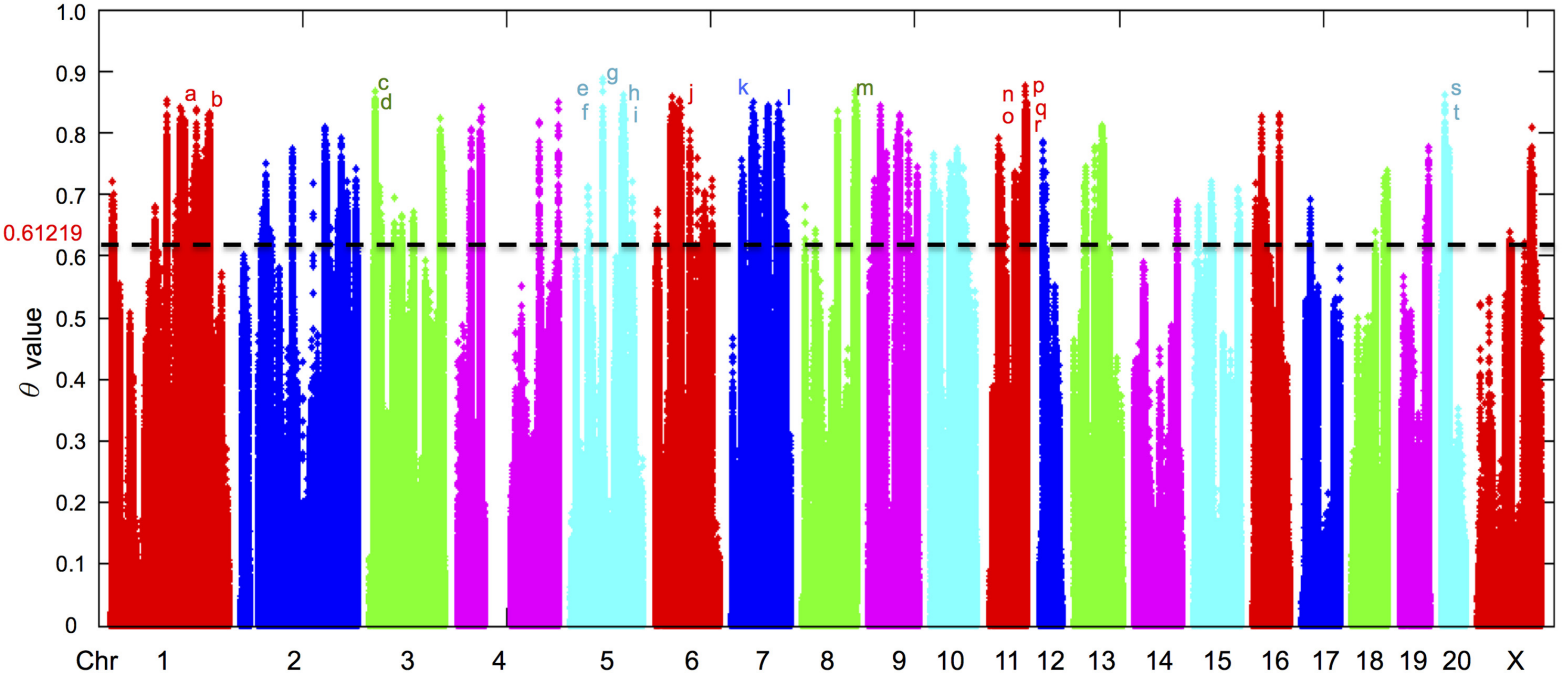
Distribution of SNPs throughout chromosomes. The distribution of SNPs is plotted based on the ‘θ’ value along chromosomes 1 to 20 and X. The red line at 0.61219 indicating the cutoff for significance at p<0.00001. Examples of genes contain the high ‘θ’ value of SNPs are labeled with alphabets in different colors responding to the color of each chromosome. a) *Fam168a*; b) *Olr126;* c) *Fcnb*; d) *Rxra; e) Mkln1;* f) *Epb41l4b*; g) *Sevp1;* h) *C8b*; i) *Dab1*; j) *Asap2*; k). *Syt1; l)*. *Denn3;* m). *Mir138-1;* n). *Klh124;* o). *Vpreb1*; p). *Ppm1f;* q). *Hic2;* r). *Yeats2; s) Stk19; t) Psmb9*. The width of the scatter plot is proportional to the size of chromosomes. There are 930 significant genes.

The genome wide average *Fst* estimated within or across replicates is given in Table 1. These statistics estimate the overall level of genetic differentiation between and within replicates, which in the absence of selection would be due to genetic drift. Signatures of selection (SS) were identified based on the intra-class correlation (θ) method of estimating *Fst* and calculated from a running average over 7 adjacent loci, the Genome Wide significance (GWp) of which was determined from 100,000 genome wide permutations (see Methods-*Detecting Signatures of selection (SS)*). There were 930 genes with θ values where GWp < .00001 (shown in S1 Table). A Manhattan plot of θ values with critical cut off and top-scored genes is illustrated in Fig 2.

**Table 1:** Alleles fixed or lost and Fst within and across replicates**.

	Fixed and Lost	
Replicate	Within Replicate	Across Replicates
1	688,080	418,177
2	592,328	
	Fst	
Replicate	Within Replicate	Across Replicates
1	0.20	0.08
2	0.19	

** Fixed and Lost indicates respectively that the non-reference allele frequency was 1 or 0.

### Landscapes of the SS

**Fig 3:**
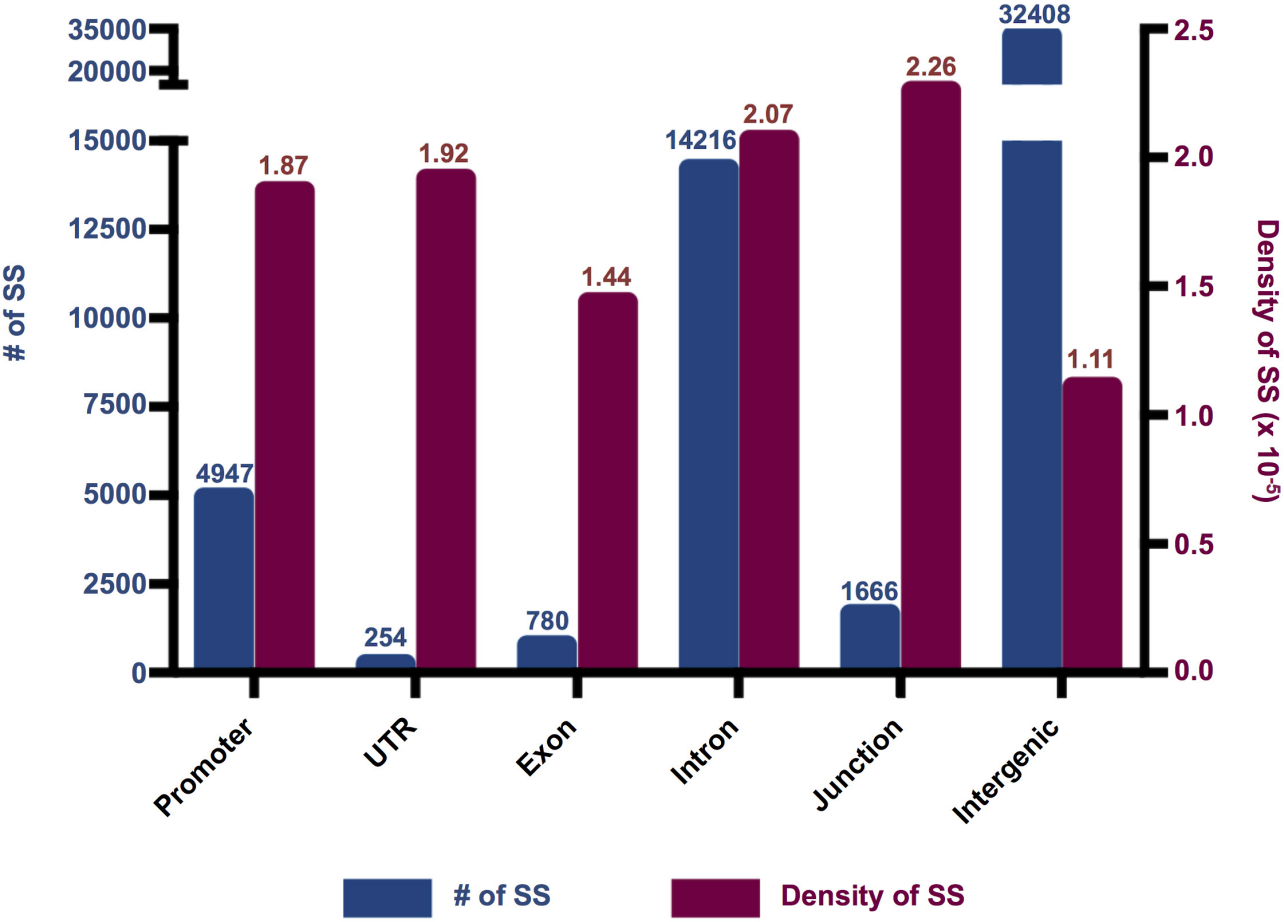
The number and density of SS observed in both replicates in gene regions throughout the genome. The total number of SS that passed the critical ‘GWp’ cutoff is shown in the blue dot line (scaled in left Y-axis). The density of SS in each region is plotted as red bar graph (scaled in right Y-axis). Density represents the total number of SS in a defined gene region divided by the total positions in that region. Note that although the number of SS in junctions is lower than introns, the density in junctions is the highest among all regions due to the fact that the total size of junctions is much smaller than the total size of introns.

Without accounting for size, the occurrence of SS in the defined gene regions (Methods-*Annotation of Data by Gene and Region*, illustrated in Fig 3), indicates that the greatest number of SS occurred in promoters and intronic regions, which were 7 and 15 times more abundant than those in exons, respectively. When adjusted for the size of the gene region (Fig 3), the greatest densities (SS/Mb) were observed in the intron-exon junctions, UTRs and promoters.

**Fig 4:**
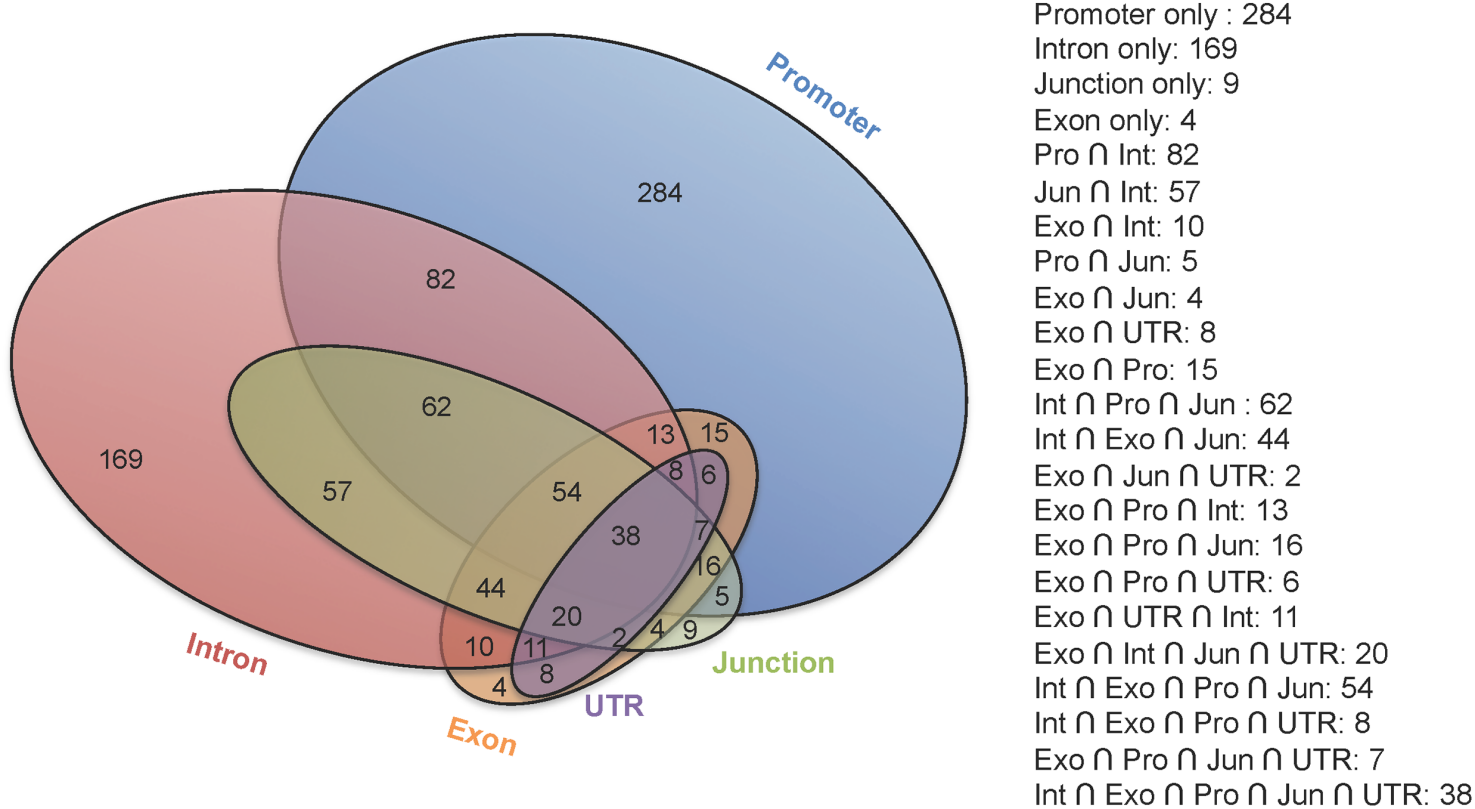
The number of genes that contain SS in different gene regions. The Venn diagram shows the number of genes that contains SS with ‘θ’ > 0.61219, corresponding to a GWp< .00001 in single or multiple gene regions. Each color circle represents one gene region, i.e. Blue: promoter, Red: intron, Green: junction, Orange: Exon, Purple: UTR. The number in each “section” within circle(s) is number of genes, e.g. 284 genes that contain SS only in the promoter, while there are 82 genes that have SS in the intron and promoter regions but not in other regions. The diagram illustrates that a large number of genes contains SS in single region of the gene. However a number of genes contain SS in multiple regions as shown in the column of overlaps on the right.

**Fig 5:**
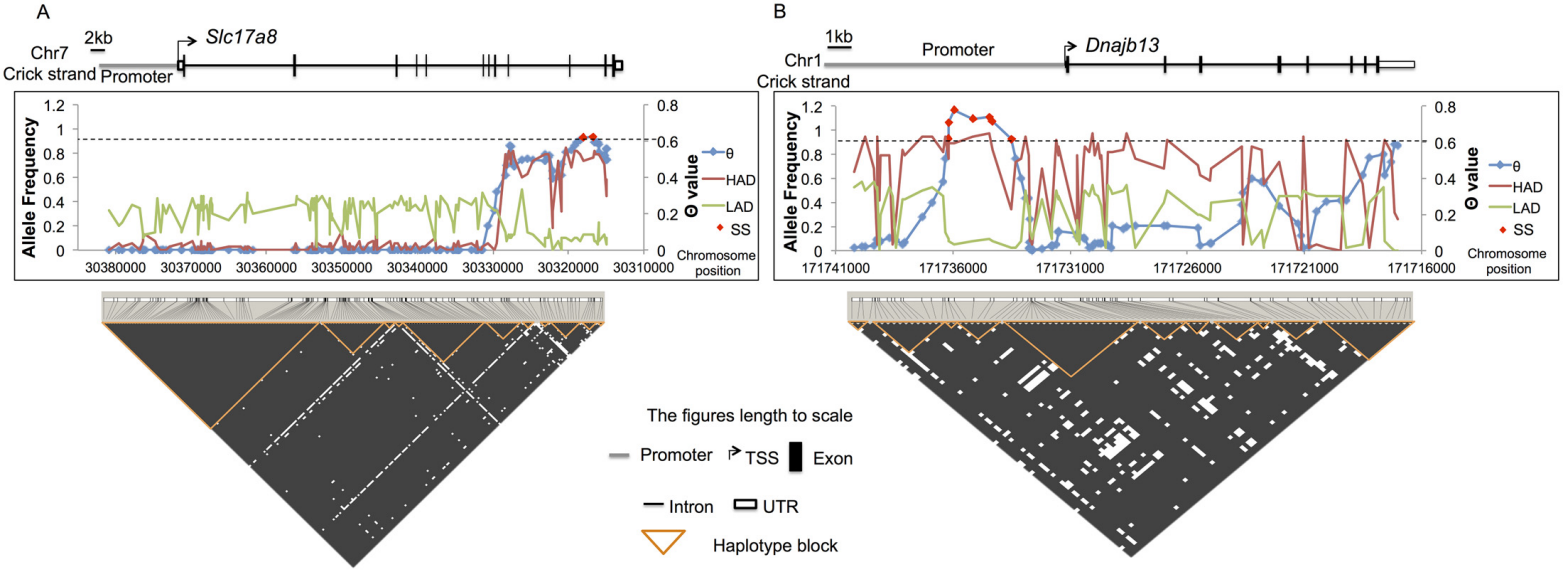
Examples of SS, Haplotype Blocks (HB), allele frequencies and θ values in two genes. (A) *Slc17a8 (ENSRNOG00000007581)* a vesicular glutamate transporter, and (B) *Dnajb13 (ENSRNOG00000017975)*, For each gene, the top panel shows the gene structure, including 10kb of promoter, transcription start site (TSS at angled arrow), exon, intron and UTRs (see figure notation in the middle of third panel). The middle panel displays allele frequencies (red, HAD; green, LAD) and θ value (blue dot-line) of all SNPs between HAD and LAD within the gene and promoter regions. Each blue dot on the θ value represents the focal SNP of the 7 SNP sliding window, with Site-specific SS highlighted as the red dot which are above the critical θ value cut off of 0.61219 (dash line). The bottom panel shows the haplotype blocks generated by Haploview [24], and where defined using the 4-gamete rule [25]. The block color represents the presence (white) or absence (black) of all 4 gametes. The presence of all 4 gametes indicates that at least one historical recombination event has occurred. A HB is defined graphically as the largest possible region (black) that is flanked on either side by at least one recombination event (white). A conservative test for recombination was used requiring that the least frequent of the 4 gametes be with a MGF of at least 9%, as such only common HB were detected. The three panels are aligned at scale with gene length.

Among the SS shown in Fig 4, the majority (50%) were located in only one region of a gene, indicating that selection operated on units within the gene (e.g. promoter, intron-exon junction), as expected based on alternative modes of gene function and expression, see also examples illustrated in Fig 5. Of the SS that mapped uniquely to a single region, the greatest numbers were in the promoters (284) and intronic regions (169) with the least in exons (4) and none in UTRs. For genes in which selection operated on more than one region, correlations between numbers of SS in those regions are given in Table 2. As expected, the correlations were generally small, except between the UTR and exon, which showed a strong positive correlation. In contrast and unexpected, a strong negative correlation was observed between number of SS in the promoter and intron regions, suggesting that selection of polymorphisms in these regions was antagonistic.

**Table 2:** Correlation between number of signatures of selection (SS) detected between regions*.

Region	Exon	Promoter	Junction	UTR	Intron
Exon	1	0.380	0.001	0.001	0.001
Promoter	-0.029	1	0.006	0.619	0.001
Junction	0.387	-0.091	1	0.001	0.001
UTR	0.567	-0.017	0.179	1	0.006
Intron	0.149	-0.456	0.363	0.090	1

*correlation below diagonal, p value above diagonal.

### Putative Functional Impacts of SNPs in SS

We identified 127 exonic SS that contained missense/non-synonymous SNPs (see SIFT analysis in Methods-*Putative Functional Impacts of SS*) in 85 genes shown in S2 Table, including *Cyp2ab1 (cytochrome P450*, *family 2*, *subfamily ab*, *polypeptide 1)*, *Scn5a (Sodium Channel*, *Voltage Gated*, *Type V Alpha Subunit)*, *Ccdc13 (coiled-coil domain containing 13)*. Some SS in promoters (S1 Table) overlapped putative transcription factor binding (TFB) sites. Examples for neuronal genes (See TFB analysis in Methods-*Putative Functional Impacts of SS*) are shown in S3 Table. In these neural-related genes, 8 out of 22 SS were associated with putative TFB and occurred at CpG dinucleotides, indicating that DNA methylation could contribute to selection. These include *Kcnf1 (Potassium voltage-gated channel; subfamily F; member 1)*, *Syt1 (Synaptotagmin I)*, and *Cck (Cholecystokinin)* (S3 Table). We detected 1575 SS in intron-exon junction regions. Ten SS are predicted to alter loci that potentially contain elements for RNA-binding proteins (RBPs; S4 Table). These include *Scn5a (Sodium channel; voltage-gated; type V; alpha subunit)*, *Syt1 (Synaptotagmin I)*, and *Ehmt2 (Euchromatic histone lysine N-methyltransferase 2)*.

**Fig 6:**
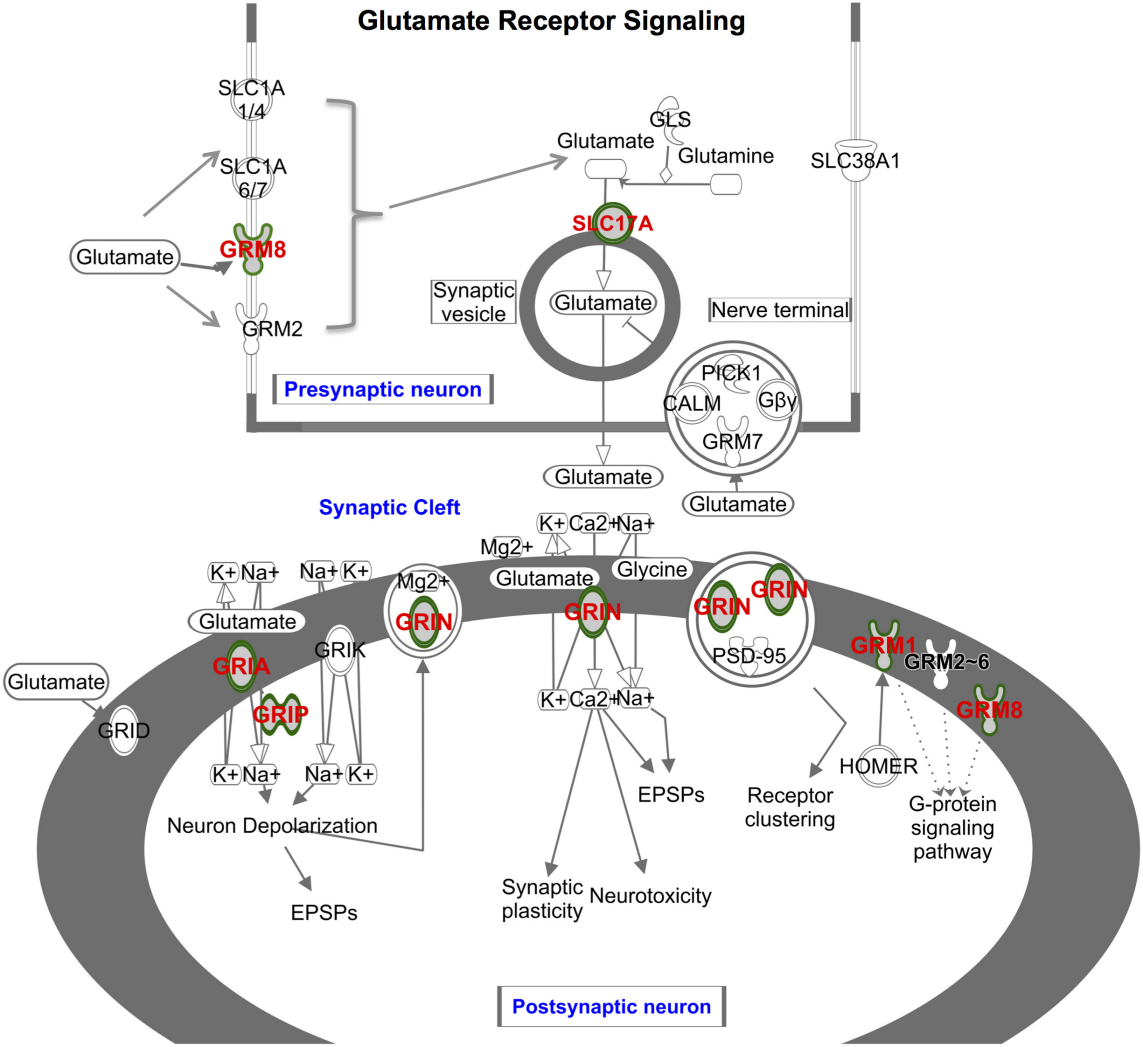
Genes with SS (color coded) within the Glutamate Receptor Signaling Pathway. These genes code for proteins that are key elements of excitatory transmission, including ionotropic NMDA (*Grin2a)* and AMPA (*Gria3)*, and modulatory metabotropic receptors mGluR1 (*Grm1*) and mGluR8 (*Grm8*). The vesicular glutamate transporters *Slc17a5*, *Slc17a8* are associated with the efficiency of the glutamate presynaptic repackaging. In addition, the glutamate receptor interacting protein 1 *(Grip1)* is involved in synaptic trafficking of AMPA and can mediate synaptic strength and synaptic memory. Misregulation of glutamate receptor signaling has been indicated in AP (see Discussion).

Among the 930 genes identified, several are involved in pathways known to be important for the regulation of alcohol consumption based on prior functional analysis and genes detected in our previous QTL mapping studies of the same animal model [11–13]. One of the most intriguing pathways is the *Glutamate Receptor Signaling Pathway*, where changes in glutamate transmission in the nucleus accumbens and amygdala have been implicated in AUD [26, 27]. Not only does this pathway contain the *Grin2a* gene (NMDA receptor subunit) in the chr10 QTL region reported by Carr *et al*. [11] in the HAD1/LAD1 lines, but also we identified five additional genes in this pathway with SS: *Gria3* (AMPA receptor subunit), *Grm1*, *Grm8* (metabotropic glutamate receptor), *Grip1* (a glutamate receptor interaction protein), and *Slc17a8* (a vesicular glutamate transporter). The proteins encoded by these genes are involved in the efficiency of excitatory communication and synaptic memory (*Grin2a*, *Gria3*, *Slc17a8)*, modulation of glutamate reception (*Grm1* and *Grm8*) and excitatory synaptic protein trafficking (*Grip1*). Their collaborative function in the pathway is illustrated in Fig 6 using IPA analysis.

Besides the glutamate system, we also found SS in a number of genes related to other transmitter systems that have been implicated or have the potential to be involved in AUD, e.g. peptidergic transmitters (e.g. *Cck*, *Pomc*), catecholamine metabolic enzyme (e.g. *Comt*), serotonin receptors (e.g. *Htr4*) and acetylcholine receptors *(Chrna10)*. Another interesting finding is that a number of genes associated with ion channels were detected. These genes serve important functions in electrical activity mediating neuronal transmission, sensitivity, and effectiveness, including Na^+^ channels (e.g. *Scn5a*, *Scn10a*), K^+^ channels (e.g. *Kcnn3*, *Kcnf1*, *Kcnc2)*, the cation channel *(Trpm8*), and calcium-activated chloride channels (*Ano10*). At the synaptic level, the genes encoding synaptic proteins e.g. *Shank2*, *Snap29*, *Syt1*, *Syt11* that mediate neuronal plasticity were also found.

We identified SS in 29 olfactory receptor genes (e.g. *Olr37*, *Olr41*, *Olr72)* that control complex olfaction as well as 24 solute carrier genes (e.g. *Slc17a5*, *Slc22a14*, *Slc35d1)* that transport essential molecules across cellular membranes, including neurotransmitters, ions, and sugar molecules. Other genes relevant to gene regulation include epigenetic modulators (e.g. *Hira*, *Ehmt2*, *Yeats2*), miRNAs (e.g. *Mir138-1*, *Mir760*, *Mir301b*) and 16 zinc finger transcription factor genes (e.g. *Zfp105*, *Zbtb8a*, *Zbtb8b*, *Zdhhc17*).

## Discussion

### Impact of Resource Population on Resolution and Ability to Fine Map

The HAD/LAD bi-directionally selected lines are uniquely suited for fine mapping for a number of reasons: a) They are one of the few replicated animal models that were initiated from the same resource population in the same generation, allowing the ability to separate signal (fixation due to selection) from noise (fixation due to random drift); b) They result from a MAGIC cross that resulted in small HB that pinpoints SS to discrete portions of a gene; c) They are derived from a long-term, bi-directionally selected population, that magnified the phenotypic and genomic differences between lines for increased power; and d) Within family selection was used to develop the lines, which maximized the effective population size and minimized the rate of inbreeding because all families contributed at least one individual to the next generation [28]. Maximizing the effective population size also minimizes the buildup of new HB [29].

Finally, while use of the *Fst* as a metric to detect SS has a number of limitations in natural populations [19, 20] due to demography, expanding populations, migration, random genetic drift, and time frame (ecological vs. evolutionary), in our experimental situation all of these variables were controlled. With the low mutation rates associated with SNPs, the *Fst* statistic is a good metric for enrichment of genes under selection, as proposed by Akey et al. [30]. However, the population specific Fst of (36) offers an alternative method of finding SS that may have more power than the average Fst used in our analysis and needs to be explored further.

### Reproducibility and Importance of Replication

Fixation of alleles can result from the combined effects of *random genetic drift* and *selection* for AP. Since alleles lost due to random genetic drift are proportional to the genome wide average *Fst* [31] (the values for which are given in Table 1), it is possible to obtain a crude estimate of the proportion of SNPs lost due to random genetic drift and also the number fixed due to selection. The model for estimating the number of SNPs fixed due to selection and lost due to drift is given by the following:

$$Lost=b_{0}+b_{1}Fst+e$$

*where*

*Lost* = the total number of alleles fixed or lost,

*b*_0_ = the intercept which is the number of alleles fixed due to selection

*b*_1_ = rate of loss of alleles due to random genetic drift

*Fst* = Wright's between population fixation index

*e* = residual error

In the absence of selection and drift (*Fst* = 0), the intercept (*b*_*0*_) is expected to be 0 because allele frequencies would remain polymorphic and constant [31]. The intercept therefore estimates the number of SNPs fixed due to selection. The least squares fit of the data to the model gives *b*_*0*_ = 255,278 ± 94,000. By comparing this number (*b*_*0*_) to the total number fixed or lost (N) within and across replicates (Table 1), one can estimate the false discovery rate [FDR = (N- *b*_*0*_)/N]. The FDR here is defined as the proportion of SNPs fixed or lost between lines that might be attributed to selection. The FDR estimated within replicates ranged from 57% (N = 592,328) and 63% (N = 688,080), while the FDR = 39% (N = 418,177) across replicates. Thus within replicates, random genetic drift accounts for the majority (>50%) of SNPs fixed and lost. When averaged over replicates, the reverse was true, the majority of fixed or lost alleles were due to selection. These rough approximations assume all loci exhibited the same initial allele frequency and were going to fixation or loss at the same rate. Nevertheless, these results illustrate the importance of replication for detecting loci under selection and minimization of false positives [21].

These results also directly address the concerns raised by Collins and Tabak [5] for lack of reproducability. Without replication, the majority of allele fixations were due to random genetic drift. This problem cannot be fixed statistically by increasing stringency for testing. For example, when analyzed by replicate, and GWp <.001, 3,698 genes were detected in Replicate-1 and 3876 genes were detected in Replicate-2, yet only 1,060 genes were verified across replicates at this GWp. *Only ~29% of genes identified by a single replicate were reproducible across replicates (71% failure rate)*. Further, increasing the stringency by decreasing GWp <.0001, resulted in 3,100 genes detected in Replicate-1 and 3,041 genes detected in Replicate-2; yet only 761 verified across replicates at this Gwp, i.e. only ~24% were reproducible, which is less than that at the lower stringency GWp. In the extreme, if only the top 1000 SS in the first and second replicates were chosen, these were associated with, respectively, 177 and 184 genes, but only 9 of these genes were verified across repliactes, which translates to a >95% failure rate. The lack of reproducibility between replicates was due to the confouncing effects of random genetic drift, not to incorrectly setting the critical Type 1 error rate.

Although lack of replication increases Type 1 error rate because the error term without replication is the mean square for variation among individuals within line, which is not appropriate as it does not include drift variance. The correct error term is the replicate x line interaction [32]. Similarly, Weir and Cockerham cautioned that even if "we were to census the entire population, our results are still affected by 'sampling' in that the particular population sampled is but one of the many possible replicates that could have arisen under the same conditions". However, even if the error variance were corrected to include drift variance, this would not correct for lack of replication. Because genetic drift is random, it is expected that a high proportion of alleles with neutral effects will drift in opposite directions [31] and it is likely that some SS could be significant within each replicate, but fixed in opposite directions in different replicates. With replication, such SS are averaged over before the *Fst* values are calculated and the SS would disappear. Thus without replication these types of SS would largely give non-reproducible results even at high stringencies, as seen here.

### Sequencing Errors, Window Size, LD and Haplotype Blocks

#### Sequencing errors

The impact of sequencing errors is mainly to increase the frequency with which heterozygotes are called. The reported error rate is 0.3% per locus for the platform we used [33], and most of those errors were false negatives [33], indicating that differences between lines were likely to be under estimated. The use of windows with running averages further reduces noise due to sequencing errors because the likelihood of multiple errors in the same window is small [34] and likely to occur in equally frequencies for each line.

#### Genotyping errors

A greater source of error is due to miss-calling genotypes due to low coverage, i.e. calling a true heterozygote a false homozygote because both alleles were not detected. With an average depth of 5, this error would occur ~6% of the time. Such errors bias the intra-class correlation (θ) downward because these errors artificially increase the estimated variance among gametes within individuals, *i*.*e*. the variance among opposite homozygotes [(0,0) *vs*. (1,1)] is greater than that between a heterozygote and either homozygote [(0,0) *vs*. (0,1)], or [(1,1) *vs*. (0,1)]. The increased gametic variance contributes to the denominator of the intra-class correlation (θ) whereas, the numerator is unbiased because, given a heterozygote, it is equally likely that either allele will be absent. Stated another way, genotyping errors due to low coverage increase the variance among genotypes, but does not bias allele frequency differences between populations.

#### Window size

Proper window size, i.e. number of SNPs in the running average, is important, but not critical to detect SS. This is because the target of selection, the quantitative trait nucleotide (QTN), may include surrounding loci due to linkage drag or hitchhiking [35]. If the window size is too large, effects of neutral loci will dilute the signal and reduce power to detect SS. If the window is too small, the signature maybe missed because the power to detect SS by including co-selected loci would be missed. By using the average R^2^ between loci to determine the window size, we ensure the use of a near optimal window size [36]. Because the amount of LD varies across the genome, if the region of selection is larger than the window size chosen, several SS will be detected in that region, but if it is too small, the SS will not be detected. Larger window sizes (15 and 31) were examined but were found to reduce power and could shift the center to a low confidence position if the larger window straddled 2 peaks (S2 Fig)

#### LD and haplotype blocks

The choice of metric to determine resolution for fine mapping and HB length is important for inference related to gene regions under selection in our analysis. The most common metric for LD is R^2^, which is a statistical measure of co-linearity between alleles at different locations in the genome. However, the use of R^2^ as a metric for SS [18], has a number of limitations. If selection starts at the time a new mutation occurs, termed a hard sweep, then selection is focused on the single HB containing the mutation, and increases R^2^ as the mutant approaches intermediate frequency, but disappears as the mutation goes to fixation. In contrast, if selection initiates based on an old mutation, i.e. the mutation has been in the population for many generations, historical recombination's will produce many different HB, each containing the favorable allele. In which case selection on the favorable allele, termed a soft sweep, may not increase R^2^ [20]. Soft sweeps usually occur due to artificial or domestication selection, while hard sweeps occur as a result of natural selection because nature is opportunistic. By starting with a MAGIC population, we ensure the existence of many HB before selection started, thus most SS are expected to be the result of soft sweeps and have little or no impact on R^2^. The distribution of R^2^ values of the HAD/LAD lines and NIH-HS-S (Fig 1A) were similar when comparing two adjacent SNPs, particularly when those 2 SNPs were close. At a distance of 7 SNPs, the R^2^ values were less similar, as would be expected with recombination by distance.

The question being addressed in our selective sweeps analysis is "what were the common chromosomal region that were co-selected across all haplotypes, i.e. completely confounded?" These common regions must have, at a minimum, been separated historically by recombination events. The 4 gamete rule (FGR) [23] addresses this question and finds where historical recombination events have occurred (Methods- *Genotypes*, *Allele frequencies*, *Linkage Disequilibrium*, *and Haplotype Blocks*). The FGR only depends on the presence of all possible gametic combinations for 2 alleles at 2 loci to occur. This procedure is the same as the recombination break point analysis proposed by Xu [37, 38] for QTL mapping with high density genotyping. While HBs defined by the FGR represent the minimum possible units of selection, to be conservative, we increased the stringency of the FGR to require a more common minor gamete frequency (MGF = 9%), which resulted in a near continuous spine of HB across most genes (Fig 5). Wang and associates [23] concluded that in humans, use of MGF > 5% is expected to find common, highly conserved, block structures given their antiquity. As such, our HB are common and larger in size than when strictly defined, but nevertheless were observed to be adequate to fine map the SS. Because the base population consisted of a multi-parent advanced generation inter-cross (MAGIC) [39–42], the multi-way crosses break up HB from the start because all four combinations of alleles possible at two loci maybe present among the parent lines (see S1A–S1C Fig) and satisfies the FGR [43] for many loci.

### Landscape of SS

#### Gene regions of SS

The greatest number of SS occurred within the intron regions (14,216), while the least occurred within UTRs (254) (Fig 3). A large number of SS were also found within the intron-exon junction regions, which potentially regulate RNA modification, as well as mRNA splicing, localization and translation [44, 45]. Several intron-exon junction SS were predicted to alter binding sites for RNA binding proteins, including splicing factors that could impact post-transcriptional gene regulation (S4 Table). Furthermore, intronic variants alter gene splicing, which has been associated with AUD [46, 47]. The large number of SS in promoters also points to the importance of transcriptional regulation, as several promoter SS were predicted to alter the binding of transcription factors between the HAD and LAD lines (S3 Table).

When adjusted for length (SS/Mb), the density of SS in the intergenic regions (Fig 3) was the lowest, indicating genes and regulatory elements were the primary targets of selection. Nevertheless, intergenic regions contain functionally relevant elements such as enhancers and repetitive elements that warrant further examination.

Among the identified SS, 50% were limited to a single region of a gene (Fig 4). The greatest number of these were in the promoter (285) and intronic subdomains (169) with the fewest in exons (4) and UTRs (0), indicating that selection operated primarily on these subcomponents, not the entire gene Fig 5. These examples demonstrate that the HBs were small enough for selection to operate on different regions of the gene. This is demonstrated in *Slc17a8* (Fig 5A), where the θ statistic is at or below background levels for the promoter and the first half of gene body, but rapidly increases at the 3’ end and in *Dnajb13* (Fig 5B), where SS were concentrated in a strong signal in the medial promoter. The SS in both genes were within HBs where no overlap occurred between SS in the exon and intron regions, indicating that selection acted separately on different regions of these genes. The results suggest that differences in AP were primarily due to regulatory changes.

#### Verification of prior QTL mapping results

Our results partially confirm previous findings using QTL analysis on Chr 5, 10, 12 and 16 of HAD1/ LAD1 lines [12], and further in Chr 10 and 16 of HAD2/LAD2 [11]. On Chr 16 of HAD1/LAD1, we found seven gene regions where SS overlapped with the QTL region. These seven regions are associated with five genes including *Grin2a*, *Cyp4f18*, *Myo9b*, *Pgls* and *Fam129c*.

### Putative Functional Impacts of SS

The neuroimmunoendocrine actions of alcohol in the gut-liver-brain network are increasing in importance as a potential mechanism underlying AUD [48–51]. Although a few of these genes have been investigated for their contribution to AUD, the scope and the major players are largely unknown. We identified SS in several genes and pathways involved in acquired immunity, including six Major Histocompatibility Class II (MHC II) genes (e.g. *Hla-doa*, *Hla-dob*, *Hla-dqa1*, *Hla-dqb1*, *Hla-drb5*, *H2-eb2*). The gene NF-kappaB signaling gene (*Nfkb1*) is involved in a wide range of immune and stress response pathways. We also identified genes with SS in the *Corticotropin Releasing Hormone Signaling pathway* which, taken together, support and provide new candidates in stress-peptide related AUD [52–54].

Excitatory and inhibitory neuronal communication pathways are directly involved in neuronal plasticity and brain adaptation, and are key to developing AUD [55, 56]. Among these, the *Glutamate Receptor Signaling Pathway* is pivotal not only in glutamate transmission, but is also critical in long term potentiation (LTP) and synaptic plasticity. It has been implicated in alcohol drinking or addictive behaviors [56–58]. Among the 6 genes in the pathway, the NMDA receptor subunit gene *Grin2a* has been associated with susceptibility of drug abuse [59, 60]. *Grin2a* contains 88 SS within the intronic regions. In a primate study [57], alternative splicing of AMPA receptor subunits *Gria3* in the prefrontal cortex was found following chronic alcohol self-administration [57]. Additionally, expression levels of *GRIA3 flip* and *flop* mRNAs were positively correlated with daily ethanol intake and blood ethanol concentrations [58]. These *flip* and *flop* variants alter the kinetics of this glutamate channel [57, 58]. *Gria3* contained 37 SS within the intronic regions. Possible effects of SNPs within the intronic regions include: alternative splicing, differential binding of transcription factors to intronic regulatory regions and epigenetic gene regulation of the AMPA gene subunit. The mGluR1 (*Grm1*) receptor has been shown to function in the brain reward system in the nucleus accumbens (NAc) regulation of alcohol intake [61]. In addition, we found 7 SS in the glutamate receptor interacting protein 1 gene *(Grip1)*. *Grip1* mediates LTP by strategically positioning the AMPA receptor towards the dendritic spine postsynaptic position. Identification of the *Grip1* receptor as a putative gene in AP is unique and has not been previously studied in this context.

Nervous system ion channels directly influence the electrical activity and efficiency of neuronal transmission. Among the potassium channel genes identified, *Kcnn3* encodes the KCa2.3 channel, which controls neuronal excitability and synaptic plasticity. This gene has been implicated in alcohol, nicotine, cocaine, and heroin abuse [62, 63]. In a mouse study [63] using an interspecific cross between C57BL/6 and DBA/2 mice with diverse drinking patterns, *Kcnn3* activity negatively modulated voluntary and excessive alcohol consumption. Low transcription levels of *Kcnn3* in the NAc were associated with higher alcohol intake. Inhibition of the KCa2 channels in the NAc of these mice may also increase alcohol consumption [63]. Our results support *Kcnn3* as a gene involved in AP.

Synaptic plasticity underlies brain adaptation and, has been shown to be disrupted in AUD [56]. We found intronic SS in the synaptic gene, *Syt1* encoding synaptotagmin, a protein which regulates presynaptic vesicular release. DNA methylation of the related gene *Syt2* in the medial prefrontal cortex (mPFC) has been linked to escalation of alcohol drinking [64] while inhibition of *Syt2* expression in the mPFC increased aversion-resistant alcohol drinking [64]. Another synaptic protein gene found in that group, *Shank2*, is a member of the Shank family that functions as a molecular scaffold in the postsynaptic density (PSD). *Shank2* attaches metabotropic glutamate receptors (mGluRs) to an existing pool of NMDA receptors critical in modulating excitatory transmission. These genes encode proteins that interact with the *Glutamate Receptor Signaling Pathways* discussed earlier and can form a sophisticated network with the potential to address AP behavior.

We also found SS in the *Cck* gene *(Cholecystokinin)*, which is a peptide hormone that stimulates digestion of fat and protein in the gastrointestinal system. This hunger suppressant has been shown to induce tolerance to opioids and plays a role in withdrawal. Chronic alcohol intake has been associated with increased sensitivity to *CCK8* [65].

Signatures of selection were found across all regions of the Catechol-O-methyltransferase gene (*Comt*), which encodes an enzyme involved in the metabolism of dopamine, adrenaline and noradrenaline. Elevated expression of COMT has been associated with AUD in a male Czechoslovakian population, which is linked to a Val158Met amino acid substitution in COMT [66]. In a Finnish population, the high (H) and low (L) activity COMT allele frequencies were compared and the L frequency was found to be significantly higher among alcoholics when compared with controls [67]. These results indicate that the COMT polymorphism contributes significantly to the development of late-onset AUD.

The neurotensin receptor 2 gene (*Ntsr2)* has two SS in intron 2 and has been implicated in alcohol dependence with conduct disorder and suicide attempts in humans [68]. In Dick *et al*’s study, two significant SNPs in intron1 of *Ntsr2*, rs12612207 and rs4669765, showed evidence for association with alcohol dependence [68]. Although the direct biological function of *NTSR2* in alcohol dependence is still unclear, previous studies reported the potential function of the neurotensin-containing pathway in linking the hippocampal and mesolimbic dopamine systems in response to drug addiction [68].

A number of genes that have been associated with human AUD, e.g. GABA-A receptor *GABRA2* [69], were not found in our analysis, although some suggestive differences were present in one HAD/LAD replicate.

In conclusion, this study revealed that AP exhibits a polygenic architecture, consistent with a complex trait and indicates that efforts to concentrate on single genes, or a few genes with large effects, are likely to result in only a small fraction of the genetic variation in the trait [2, 69, 70].

## Materials and Methods

### Breeding and Selection of Paired-Lines

Two replicate HAD and LAD lines were initiated from the N/NIH HS heterogeneous stock [71] (i.e., with different parents in two different colonies) and selectively bred using within family selection and a rotational breeding program [10]. During selection both lines were given free access to food, water and a 10% (v/v) ethanol solution. The selection criteria for the HAD lines were consumption of at least 5.0 g of ethanol/kg body weight/day, with an ethanol to water ratio of at least 2:1, while LAD rats were required to drink less than 1.5 g/kg/day with an ethanol to water ratio of less than 0.5:1. Bi-directional selection for AP was repeated continuously for 30 generations, followed by generations of selection that were interspersed with relaxed selection. Sixty generations were completed at the time of sampling, of which 40 were for and against AP in each replicate.

### DNA Isolation and Quality Control (QC)

Spleen samples from 10 alcohol naïve rats (5 males and 5 females) of each line and replicate were collected and stored at -80°C. DNA was isolated using Gentra Puregene Kits (Qiagen, Redwood City, CA, USA). About 25 mg of spleen was homogenized in cell lysis solution, followed by proteinase K digestion, RNase A incubation, and DNA precipitation as described in manufacture’s protocol. DNA purity (260/280 ratio) measured 1.79–1.94, with the majority >1.85. The DNA yield was more than 200 μg in most samples. One of the samples was removed from the analysis as genetic testing indicated its ID was incorrectly recorded.

### Sequence Assembly and Variant Calls

Individual DNA from each rat was sequenced in the IU Center for Medical Genomics using an ABI SOLiD 5500xl platform using a 35/75 bp paired end fragments protocol in the first replicate and 75bp single end protocol in the second. We reached an approximate depth of 5x per sample and 50x across each line within a replicate. Reads were mapped to the reference genome (Rattus_norvegicus.Rnor_5.0.71) using LifeScope (http://www.lifescopecloud.com). Duplicate reads were removed using Picard (http://broadinstitute.github.io/picard/), and reads with mapping quality < 8 were also removed. Variants (SNPs) were called using GATK multi-sample variant calling [8]. Reads with coverage greater than 4 times the expected read depth across all samples were removed as possible copy number variants or other genome duplication events. A 0.3% sequencing error rate was assumed [72] and screened for by combining all samples and if the total number of SNPs at that location was less than 0.3% of total reads, the location was removed.

### Genotypes, Allele Frequencies, Linkage Disequilibrium, and Haplotype Blocks

Allele frequencies in each line and replicate at a given locus were estimated as the total number of non-reference (NR) alleles present across samples within a replicate to the total reads at that locus (N). For estimating LD and HB, genotypes were called for each individual at each polymorphic locus. Gametic phase disequilibrium (or Linkage disequilibrium, LD) was estimated pair wise between all polymorphic loci on the same chromosome as the coefficient of determination (R^2^) using the iterative method [33]. Only loci with a minor allele frequency (MAF) > 10% were used to calculate LD to avoid issues related to rare alleles. In addition, following the methods of Wang and associates [73], haplotype blocks (HB) were defined as chromosomal regions flanked by at least one historical recombination event, termed the Four Gamete Rule (FGR) [23], and as implemented by Haploview [43]. In brief, blocks [25] are searched from the start of a region by sequential addition of the next locus, the number of unique gametes between the initial and last locus are counted. When all four gametes are observed between the last locus and any of previous loci in the block, the position of the last locus is regarded as the putative starting point of a new block, and the block size is determined as the sequence length between the start and end positions. The only explanation for observing all four gametes between a pair of loci is the occurrence of at least one historical recombination event [23]. While strict use of the FGR would require only one recombination event to infer an HB, for purposes of examination of HB as units of selection, a more conservative Minor Gamete Frequency (MGF) was used. Increasing the MGF results in larger HB. The MGF was therefore increased until a near continuous spine of HB resulted as defined and visualized by Haploview. A continuous spine results when nearly all SNPs are contained in HB and a new HB starts where the previous ends. This process of finding a continuous spine will tend to only find common HB, which for our purposes is the desired outcome. However, for purposes of comparison of HB size to the NIH-HS-S (see Methods-*Estimation of allele frequencies in the NIH-HS parent lines*) we used a MGF of 6.25% (1/16) because allele frequencies in that population were coarse with the smallest expected non-zero MGF = 1/2N for N = 8 lines.

### Estimation of Allele Frequencies in the NIH-HS Parent Lines

The initial allele frequency was estimated from the parent lines that composed the NIH-HS. The 8 parent lines of the NIH-HS i.e., ACI, BN, BUF, F344, M520, MR, WKY and WN, were recently sequenced (Rat Genome Sequencing and Mapping Consortium, 2013). We downloaded the genomic data (ERP001923) from the NCBI Sequence Read Archive (SRA) and mapped the sequence to the reference genome used in this study (Rattus_norvegicus.Rnor_5.0.71). Our results were essentially the same of that of the Consortium, except we detected that some SNP loci were still segregating within some lines, although the differences were minor. Allele frequency was estimated within each line and locus as the number of SNP reads at that locus over the read depth at that locus. These were then averaged over the 8 parent lines. We refer to the "*in silico*" NIH-HS as the NIH-HS-S line.

### Detecting Signatures of Selection (SS)

Signatures of selection (SS) were detected as excessive differentiation measured by Wright's *Fst* statistic [23], but estimated as the intra-class correlation due to between line differentiation (θ), based on a simple extension of the method presented by Weir and Cockerham [15]. Weir and Cockerham (1984) showed that it is possible to define 'F'-statistics based on the method of moments from the analysis of variance. In a nested analysis, variance components can be estimated for 1) between population, 2) within population between individuals, and 3) between alleles within individuals, and 4) total variation. Based on various ratios of these they defined: F, f, and θ which correspond to Wright's Fit, Fis, and Fst. Because Fst, estimated as θ, was defined as a ratio of the between population variance component to the total variance, we refer to this statistic as an intraclass correlation as defined by Fisher [74]. The *Fst* statistic is usually found in running windows of arbitrary size [17]. We chose a window size based on the coefficient of determination (R^2^) between loci as suggested by Weir and colleagues [34]. The quantity (1- R^2^) is the proportion of the covariation between loci that is independent of linkage. With an R^2^ < 50%, the majority of covariation is independent. Thus at a minimum, the distance between loci should be greater than R^2^ = 50%. We chose a more conservative critical value of R^2^ = 25% (LD_25). The median number of SNPs between loci associated with the LD_25 was used as the size of the sliding window for the *Fst* statistic. Details of the method are as follows

Following Weir and Cockerham [15], the *Fst* statistic was estimated based on the following linear model where Y is the presence or absence of the SNP at the q^th^ locus in the m^th^ gamete

$$Y_{ijkm}=\mu +L_{i}+R_{(i)j}+S_{(ij)k}+G_{(ijk)m}$$ (1)

for the i^th^ Line (i = 1,2), of the j^th^ Replicate (j = 1,2), in the k^th^ Sample (k = 1,10), and m^th^ gamete (m = 1,2) The expected mean squares for this model, in terms of drift variance, was given by Muir [32]. From the analysis of variance, variance components for each term were estimated for each locus containing a polymorphic SNP. The *Fst* statistic was estimated as θ in sliding windows of size w following Equation 10 from Weir and Cockerham [15] for multiple loci as

$$\theta_{\left(q\right)}=\frac{\sum_{q=1,w}\sigma_{L}^{2}{}_{q}}{\sum_{q=1,w}\left(\sigma_{Lq}^{2}+\sigma_{R(L)q}^{2}+\sigma_{S(RL)q}^{2}+\sigma_{G(RLI)q}^{2}\right)}$$

Statistical Significance was determined by genome wide permutations [75] whereby the design classifications were randomized onto gametes, following which the statistic was evaluated at all loci across the genome and the largest value recorded. This process was repeated 100,000 times. Among the ordered 100,000 largest values, the genome wide critical *θ* value (GWp) corresponded to the p% largest ordered *θ* value. Because all SNP locations across the genome were included in the permutation analysis, the critical value is automatically adjusted for multiple comparisons. As an additional level of caution, permutations were also conducted at the individual level, where the design classifications were randomized onto individuals, with their complete set of genotypes across loci. This method of permutation preserves the covariance structure among the gametes within individuals. These were also permutated 100,000 times and the GWp estimated in the same manner. The larger critical value from the two permutation methods was used as the GWp.

### Manhattan Plots

To give a global perspective of the genome-wide magnitude and spatial distribution of SS, we created a Manhattan plot based on the ' θ ' values on Chrs 1–20 and the X. The plot was generated using MATLAB 8.3 (The MathWorks, Inc., Natick, MA, USA).

### Annotation of Data by Gene and Region

Significant ' θ ' values in windows of running averages were annotated by gene regions. Gene locations and regions were based on the current Ensemble annotation of the rat genome (http://useast.ensembl.org/info/genome/index.html) version 5.0 and included: exon, untranslated region (UTR) and intron. The promoter region was defined as a 10kb domain upstream of the transcriptional start site in order to capture the proximal promoter, closely-linked enhancer elements and regions subject to epigenetic regulation.

Critical *cis*-acting splicing elements within the intron include the 5’ splice site, the branch point, the polypyrimidine tract and the 3’ splice site were included in the intron-exon junction fragment. Although the branch point is most commonly found within the last 40 nucleotides (nt) of the intron, its position is highly variable and has been mapped up to 400 nt away from the 3’ splice site [76, 77]. Analysis of several hundred human exons indicates that the majority of branch points are located within 150 bp of the 3’ splice site [78]. To ensure we captured the majority of branch point sequences, we divided introns into two components: a) intron-exon junction regions (JR) 150bp upstream and downstream of each exon for multi-exon genes and b) intron-non junction (INJR) regions, which capture intronic sequences that extend beyond 150 bp from the exon. The JR include some, but not necessarily all alternative splice sites, while the INJR may pick up additional alternative spice sites and other non-coding regulatory factors, such as enhancers and non-coding RNA binding elements.

### Putative Functional Impacts of SS

Effects of SS in the exonal region were determined by the Ensembl Variant Effect Predictor (VEP): http://www.ensembl.org/info/docs/tools/vep/index.html [79], which uses the *Sorting Intolerant From Tolerant* (SIFT) software (http://sift.jcvi.org) [80] to predict the effect of SNPs in protein function [81]. The current version of Ensembl VEP uses rat genome Rnor_6.0 assembly, we therefore convert our data from Rnor_5.0 to Rnor_6.0 using Ensembl Assembly Converter (http://useast.ensembl.org/common/Tools/AssemblyConverter) before uploading data to VEP. To predict the effects of promoter SS on altering putative transcription factor binding, we retrieved 20 bp of flanking sequence (10bp on each side of the SS; 21bp total) from the RGSC Rnor_5.0 rat genome assembly, and put the sequence into PROMO (http://alggen.lsi.upc.es/) [82]. The HAD and LAD sequences were analyzed separately for putative transcription factor binding sites and additions or loss of CpG dinucleotide. For the prediction of RNA binding factors on junctional SS, similar to promoter SS, 10bp of flanking RNA sequence (converted T to U) on each side of the junctional SS were input to http://cisbp-rna.ccbr.utoronto.ca/TFTools.php [83, 84], using the model of PWMs-Log Odds, and threshold of 6. For genes expressed from the Watson strand, we used the published sequence; for genes expressed on the Crick strand, we used the reverse complement. The HAD and LAD sequences were analyzed separately for putative RNA factor binding sites.

Pathways of biological interest were defined and visualized using QIAGEN’s Ingenuity Pathway Analysis (IPA; Qiagen, Redwood City, CA, USA, www.qiagen.com/ingenuity). We did not do an enrichment or over-representation analysis common in RNA-seq studies because, as opposed to an RNA based expression analysis where all pathway members have the opportunity to be expressed, at the DNA level not all genes in a pathway may have had the opportunity to be selected upon due to lack of new mutation within those genes, or intolerance of those genes to new mutations, i.e. pathway analysis would fail to detect pathways under selection if selection did not have an opportunity to function because no new mutations were present to act upon. As such, pathway analysis is expected to exhibit a large number of false negatives and should not be used to prioritize pathways found via number of genes with SS. But, it is also reasonable to assume that multiple genes from a crucial pathway could be under selection and contribute to collaborative function.

## Supporting Information

S1 Fig Gametic phase disequilibrium (LD). (A)Two way inbred cross. With no recombination, in the F2 the effect of the A locus is completely confounded with the B and R^2^ = 1. (B) At equilibrium R^2^ = 0 and all 4 gametes are equally frequent. (C) A MAGIC cross of inbreds. Gametic Phase and Zygotic Phase Equilibrium can be achieved immediately in the F1, LD = 0 (R^2^ = 0) because a MAGIC cross takes advantage of historical recombination's in some inbred lines that occurred prior to crossing during the formation of the inbred lines themselves.
(TIFF)

S2 Fig Impact of window size on positions of signatures of selection. (TIFF)

S1 Table Significant genes and regions containing SS with θ values where GWp < .00001. (XLS)

S2 Table Exonal SS that contained missense/non-synonymous mutations. (XLS)

S3 Table Neural genes with SS in transcription factor (TF) binding sites of the promoters. (XLSX)

S4 Table Genes with junctional SS that are predicted to alter binding of RNA binding proteins. (XLSX)
