# Supplementary figures and images for: High Resolution Genomic Scans Reveal Genetic Architecture Controlling Alcohol Preference in Bidirectionally Selected Rat Model
Source: PLoS Genet. 2016 Aug 4;12(8):e1006178. doi: 10.1371/journal.pgen.1006178 (PMC4973992; doi:10.1371/journal.pgen.1006178)

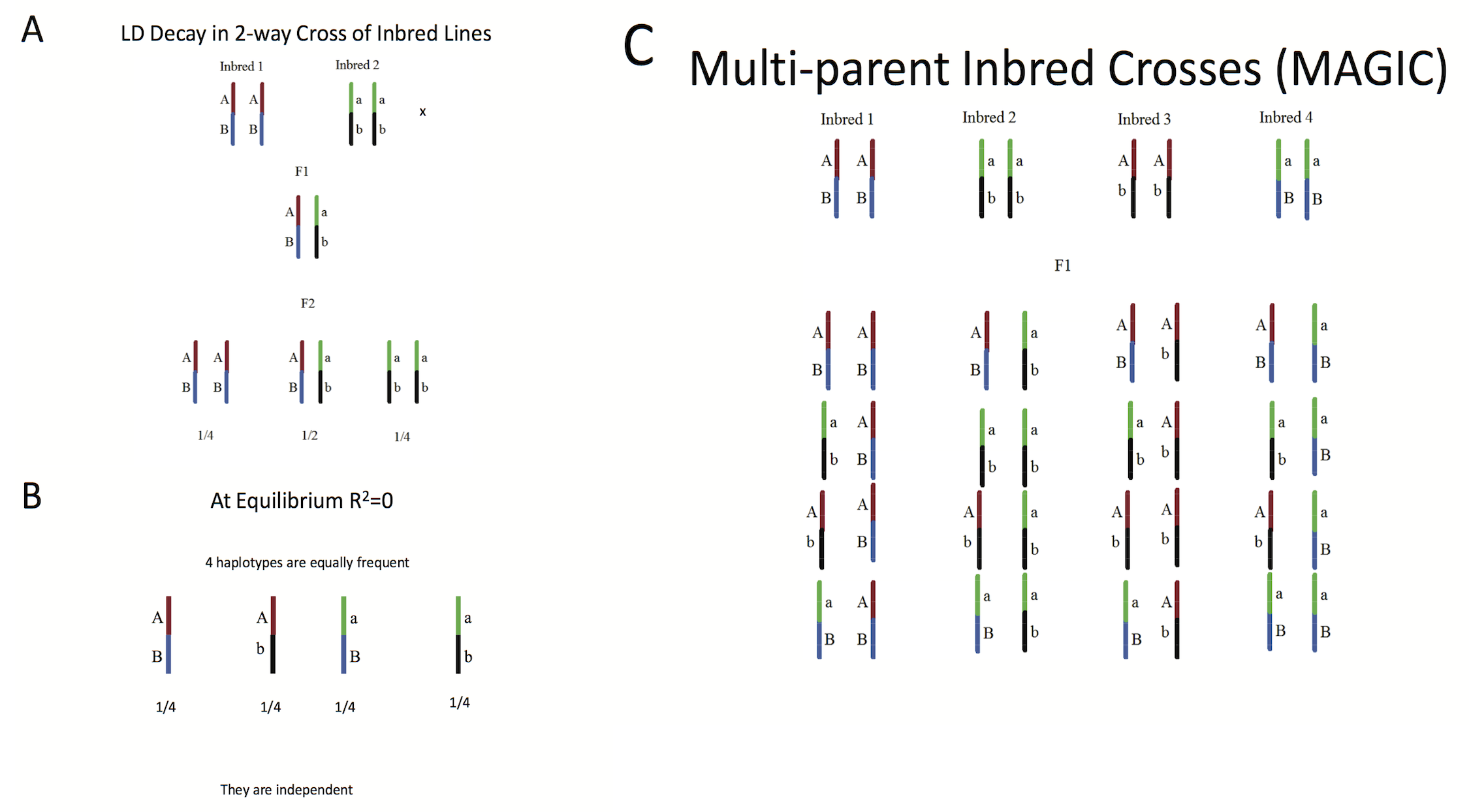

Supplement: S1 Fig — (A)Two way inbred cross. With no recombination, in the F2 the effect of the A locus is completely confounded with the B and R2 = 1. (B) At equilibrium R2 = 0 and all 4 gametes are equally frequent. (C) A MAGIC cross of inbreds. Gametic Phase and Zygotic Phase Equilibrium can be achieved immediately in the F1, LD = 0 (R2 = 0) because a MAGIC cross takes advantage of historical recombination's in some inbred lines that occurred prior to crossing during the formation of the inbred lines themselves. (TIFF) [file pgen.1006178.s001.tiff]

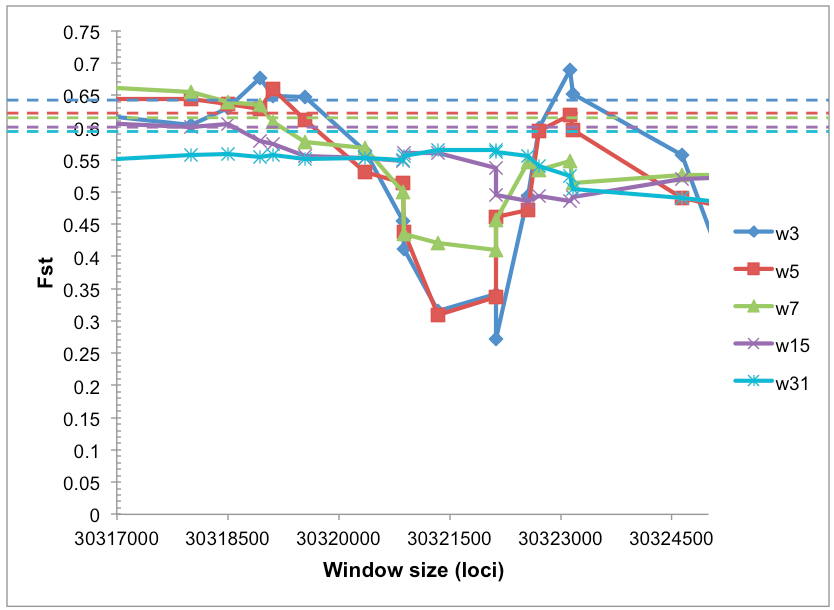

Supplement: S2 Fig — (TIFF) [file pgen.1006178.s002.tiff]
